# Novel human antibody therapeutics: The age of the Umabs

**DOI:** 10.1002/biot.200800110

**Published:** 2008-10

**Authors:** Sigrid R Ruuls, Jeroen J Lammerts van Bueren, Jan G J van de Winkel, Paul W H I Parren

**Affiliations:** 1 Genmab, Yalelaan 60, 3584 CM UtrechtThe Netherlands; 2 Immunotherapy Laboratory, Department of Immunology, University Medical CenterUtrecht, The Netherlands

**Keywords:** Antibodies, Cancer, Human, Therapeutics

## Abstract

Monoclonal antibodies represent a major and increasingly important category of biotechnology products for the treatment of human diseases. The state-of-the-art of antibody technology has evolved to the point where therapeutic monoclonal antibodies, that are practically indistinguishable from antibodies induced in humans, are routinely generated. We depict how our science-based approach can be used to further improve the efficacy of antibody therapeutics, illustrated by the development of three monoclonal antibodies for various cancer indications: zanolimumab (directed against CD4), ofatumumab (directed against CD20) and zalutumumab (directed against epidermal growth factor receptor).

## 1 Science-based discovery

Monoclonal antibodies (mAbs) have become a critical component of clinical treatment regimens for a variety of indications. Their application ranges from cancer, inflammation, cardiovascular diseases, and transplant rejection to infectious disease. Drugs like trastuzumab, rituximab and inflix-imab have demonstrated that mAbs can be used as highly specific therapeutics, able to elicit significant and prolonged clinical responses. Since the introduction of the first US Food and Drug Administration (FDA) approved therapeutic mAb (Ortho-clone OKT3, muromonab-CD3) into the clinic, 21 more antibodies have been approved for use in humans, and many companies have antibody products well advanced in clinical development.

Even though mAbs have brought major advances to the clinical practice, it is clear that there remains room for improvement. Indeed, subsets of patients are not responding to initial antibody treatment, or become resistant to (re) treatment. Research on the identification of biomarkers that allow the prediction of responding patients has shown that besides target expression and state, biomarkers such as antibody-binding Fc receptors and immune-modulatory molecules also impact clinical outcome.

Moreover, antibodies can exert their action in a variety of ways, ranging from target modulation, neutralization of soluble targets (such as cytokines), disruption of ligand-receptor interactions and influencing cell signaling, to engagement of immune effector functions such as antibody-dependent cellular cytotoxicity (ADCC) and complement-dependent cytotoxicity (CDC). The mechanisms of action of a therapeutic antibody strongly influence its application and clinical potential. Especially in oncology, it is observed that successful mAbs can exert multiple mechanisms of action. In cancer therapy, a therapeutic agent needs to (at least) control malignant cell growth, and preferably be able to kill and eradicate the target cells. mAbs have, in this respect, a double benefit: not only do they have the ability to directly interfere with cell growth, they can also specifically activate the immune system to induce cell lysis or phagocytosis.

Applying this knowledge in the discovery of new therapeutic mAbs makes it possible to improve the panels of antibodies generated, and to select the best candidates for clinical development. At Genmab, we use this science-based approach to generate fully human antibodies for a variety of indications, with a strong focus on oncology. We describe here how this has resulted in the development of three mAbs that are now in Phase III clinical trials in various cancer indications: zano-limumab (directed against CD4), ofatumumab (directed against CD20) and zalutumumab (directed against epidermal growth factor receptor, EGFR).

## 2 Tackling immunogenicity

Like other therapeutic proteins, antibodies can be immunogenic, and the generation of anti-antibody responses may pose safety concerns. In addition, anti-antibody responses can influence pharmacokinetics, or reduce efficacy of the injected antibody through neutralization.

**Figure 1:**
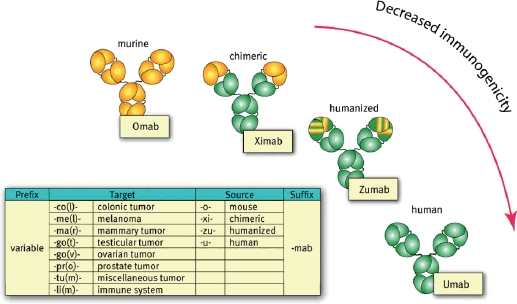
Classification of therapeutic antibodies in cancer. Progress in genetic engineering has facilitated the development of fully human therapeutic mAbs. Original mAb technologies yielded murine (and in some cases rat) molecules. Chimeric antibodies are genetically engineered mAb with murine variable regions (VL and VH) and constant regions derived from a human source. H umanized therapeutic mAb closely match the human germline sequence except for CDR, which are of murine (and occasionally rat) origin. The inset table explains the nomenclature of therapeutic antibodies in cancer indications, according to the system of International Nonproprietary Names (INN).

The first therapeutic mAbs were of mouse origin (indicated by the suffix -omab in INN nomenclature; see Fig. 1 for more explanation on antibody nomenclature), and frequently caused infusion reactions that could be as severe as anaphylactic responses [1]. To tackle this problem, molecular biological approaches were used to replace part of the rodent antibody sequence for human sequences [2, 3], resulting in chimeric or humanized molecules (suffixes -ximab and -zumab in INN nomenclature Fig. 1). Even better, technologies now exist to generate fully human antibodies (suffix -umab). We employ transgenic mice, in which the murine antibody genes have been inactivated and replaced by human antibody genes (human Ig transgenic mice, [4]). In these mice, normal somatic hypermutation, affinity maturation and class switching occurs following repeated immunizations, resulting in high-affinity antibodies. Immunization, fusion of B cells, and hybridoma propagation use similar approaches as in classical monoclonal production, but now with fully human antibodies as the end product. The transgenic mouse platform employed includes several mouse strains, each containing DNA encoding parts of the human variable region antibody repertoire and varying human constant regions. Typically, the mice contain at least a human kappa light chain transgene and transgenes for human μ and γ1 heavy chains [4, 5]. Selection for the desired IgG subclass can be included in the antibody selection process, or alternatively the subclass can be adapted using standard molecular biological techniques in the process of developing a stable recom-binant cell line required for large scale production.

In contrast to human antibodies derived from other technologies such as phage display (when based on naïve libraries), antibodies from transgenic mice do not require any further engineering. Indeed, the available literature on the drug discovery of transgenic mouse-derived antibodies indicates that they typically move directly from lead selection into clinical development. In contrast, lead optimization of phage display-derived antibodies to improve affinity is often described as an integral part of their development (reviewed in [6]). As clinical experience with fully human antibodies derived from both these technologies is limited, it is currently unresolved whether the introduction of novel sequences during lead optimization impacts immunogenicity.

There are a number of factors that may influence the immunogenicity of a therapeutic protein. Not only structural properties of the protein itself (as discussed below), but also ‘external’ factors such as drug impurities, formulation, route of application, dose and length of treatment may influence immune responses to therapeutics [7]. In addition, patient characteristics, including genetic background, disease state and concomitant treatments, determine whether antibody responses are elicited.

The presence of so-called T helper cell epitopes in the therapeutic protein itself can contribute to its immunogenicity. In the generation of an antibody response, (foreign) proteins are taken up by antigen-presenting cells (APC), and processed intra-cellularly into small peptides that are exposed on the APC surface in the context of major histocom-patibility complex class II molecules (MHC II, in humans also known as human leukocyte antigens, HLA). These peptides, or epitopes, are then recognized by T helper cells, which are activated to proliferate and differentiate. T helper cells in turn stimulate specific B cells to produce antibodies directed against the antibody drug to produce anti-antibodies^1^. The MHC/HLA II locus is highly variable, and numerous MHC/HLA allotypes exist, which all have their own peptide specificities.

Classically, identification of T helper cell epitopes is performed by *in vitro* tests using human blood cells. Nowadays, more sophisticated *in silico* approaches exist, which are powerful tools to allow mapping of epitopes from virtually all HLA allotypes. In particular, structure-based methods have been found to reliably predict T helper epitopes [8]. We have used one such method, Epibase® [8], to investigate whether the expected low immunogenicity of fully human antibodies can be confirmed (Table 1). This method not only predicts T helper epitopes, but also indicates peptide affinities for HLA allotypes. A striking result from these analyses was that the numbers of predicted strong binding T helper epitopes of zanolimumab, ofatumum-ab as well as zalutumumab were very low indeed. Direct comparison of, for instance, ofatumumab to rituximab, a chimeric CD20 antibody that has been on the market since 1997, revealed ofatumumab to contain at least four times less T helper epitopes (Table 1) [8] .We compared our antibodies not only to chimeric or humanized products, where large differences can be expected, but also head-to-head for zalutumumab and panitumumab. The latter is a fully human antibody against EGFR, derived from another transgenic mouse platform developed by Abgenix (now Amgen) [9]. Remarkably, two times more strong-binding epitopes for HLA DRB1 were found in panitumumab compared to zalutumumab (Table 1). As expression levels of HLA DR1 are (much) higher than those of DQ and DP, binding epitopes for DR1 molecules are considered to represent the most important differentiators in immunogenicity of proteins. The transgenic mouse platform (Xenomouse®) used to generate panitumumab and the UltiMAb® platform [10] employed to generate zanolimumab, zalutumumab and ofatumumab contain differences inVH,-, D- and J-gene repertoire in a distinct MHC background. In addition, the specific strain used to generate panitumumab did not contain a Cγ1 gene (and only contained the human Cγ2 gene instead). This could have contributed to the differences found.

**Table 1:** Number of strong binding T helper epitopes (*K*_d_ <100 nM, [8]) identified by Epibase present in CD4-binding antibodies Zanolimumab and chimeric Leu3a-, CD20-binding antibodies ofatumumab and rituximab, and EGFR-binding antibodies zalutumumab, cetuximab and panitumumab. Results are split up per human leukocyte antigen class II type

Target	Antibody	DRB1	DRB3/4	DQ	DP
**CD4**	**Zanolimumab**	7	1	3	1	
	**Chimeric Leu3a**	10	2	2	0
**CD20**	**Ofatumumab**	4	0	2	2
	**Rituximab**	16	2	4	1
**EGFR**	**Zalutumumab**	3	2	3	1
	**Cetuximab**	16	2	3	3
	**Panitumumab**	7	0	2	0

The first data on immunogenicity in a clinical setting confirm that our human antibodies do not elicit strong immune responses. In a Phase II study in refractory cutaneous T cell lymphoma (CTCL), only 1 out of 47 patients receiving zanolimumab developed a titer of human anti-human antibodies that was marginally above background [11]. There was no indication that this human anti-human antibody response was neutralizing the effect of zanolimumab. In contrast, initial studies with chimeric CD4 antibodies in this indication [12] showed that already after a short-term treatment 2 out of 7 patients developed human anti-chimeric antibodies. The first results for ofatumumab showed that this molecule did not elicit human anti-human antibodies [13].

Another, very powerful, *in silico* approach to identify potential immunogenicity is the ‘collier de perles’ analysis and direct comparison of the nu-cleotide and amino acid sequences of the V domains of antibodies as provided by the IMTG database [14]. This approach provides a standard delimitation of the framework regions and complementarity determining regions (CDRs), and allows comparisons to the closest germline sequences of these regions. As an illustration of the usefulness of this approach, Magdelaine-Beuzelin *et al.* [14] analyzed a number of chimeric and humanized antibodies (cetuximab, rituximab, alemtuzumab, beva-cizumab and trastuzumab). They described an expected low percentage of identity of chimeric antibodies to the most similar human germline sequence (55–80% identity). Remarkably, humanized antibodies fell in this same range, with 72–80% identity to human germline. Antibody responses have been reported to all chimeric and humanized antibodies currently in the clinic (for a comprehensive overview, see [8]). Although the incidence of such antibody responses has certainly not been documented in all patient groups [for instance, anti-rituximab responses are readily found in autoimmune disease patients, but not in non-Hodgkin's lymphoma (NHL) patients], identification of apparent deviations from germline sequences could aid in the design and perfection of therapeutic antibodies. We have screened zanoli-mumab, ofatumumab and zalutumumab against the IMTG human reference directory (Neijssen *et al.*, manuscript in preparation), and observed that the VH and Vκ sequences of these three antibodies are remarkably close to germline. When comparing the V region sequences with the closest human sequences found, the percentage of identity ranged from 97% to 100%.

Overall, independent methods to examine immunogenicity of zanolimumab, ofatumumab and zalutumumab substantiate that the human Ig transgenic mouse platform generates fully human antibodies with favorably low immunogenicity profiles, which are expected to pose low safety risks in the clinic.

## 3 Zanolimumab: Changing CD4^+^ T cell activation and survival

The CD4 molecule is a single-chain transmembrane glycoprotein of 55 kDa that consists of four extracellular Ig-like domains and a short cytoplasmic tail. CD4 is mainly expressed on T helper cells and monocytes and serves as a co-receptor for the T cell receptor (TCR)/CD3 complex. The function of CD4 is to enhance antigen-mediated activation of T cells. This is achieved by stabilizing the adhesion between T cells and APCs (association of CD4 and MHC II/peptide complex) and enhancing signal transduction of the TCR/CD3 complex [15, 16]. With CD4 playing a key role in immunity, therapeutic research of CD4 mAbs was initially focused on inflammatory and (auto) immune diseases [17–20]. Our fully human CD4 mAb zanolimumab was assessed in Phase I/II clinical trials in rheumatoid arthritis and psoriasis. The predominant outcome of these studies was that zanolimumab was not only found safe and well tolerated, but most importantly, it induced a significant T cell depletion following repeat dosing [21]. Development of the antibody was discontinued in inflammatory indications as it did not show a benefit over approved drugs for these indications. However, the ability to particularly deplete memory type T cells [21, 22] suggested the potential of zanolimumab to treat T cell cancers (see below).

Zanolimumab (clone 6G5, [5]) was among the first fully human mAbs generated from human Ig transgenic mice. Biochemical analyses of the panel of antibodies from which zanolimumab was selected showed the strength of the transgenic mouse platform, being able to generate highly specific antibodies with binding capacities (avidity) similar to those of ‘classical’ murine mAbs.

**Figure 2:**
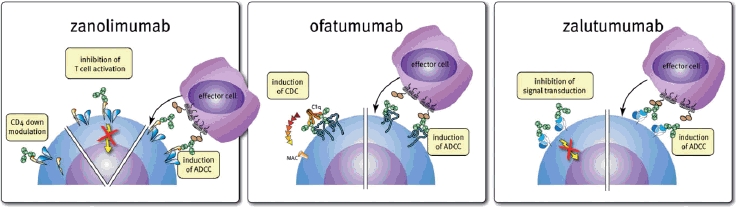
Mechanisms of action of zanolimumab, ofatumumab and zalutumumab. See text for further description.

Preclinical studies in non-human primates gave the first indications for the CD4^+^ cell-depleting capacity of zanolimumab [23]. Bolus injection of zanolimumab in cynomolgous monkeys significantly depleted CD4^+^ cells from the blood, with recovery taking up to 160 days. A detailed study was initiated to elucidate the mechanism(s) responsible for the reduction of circulating CD4 cell numbers by zanolimumab [22] (Fig. 2). Zanolimumab strongly inhibited T cell activation through two distinct, complementary pathways. One of the earliest events in the TCR signaling cascade, the phospho-rylation of the TCR chain, was inhibited by zanolimumab. Interestingly, zanolimumab binding activates signaling through the CD4-associated tyrosine kinase p56^LCK^. Herein, the antibody uncouples p56^LCK^ from the TCR, which allows the kinase to transmit direct inhibitory signals through inhibitory adaptor molecules Dok-1 and SHIP-1.

In addition, Fc-dependent effector mechanisms were studied. Although zanolimumab is of human IgG1 isotype, and is intrinsically capable of activating complement (as determined in plate-bound assays), the antibody did not bind or activate complement when bound to CD4 on primary T cells. In contrast, already at relatively low concentrations zanolimumab induced potent NK cell-mediated ADCC of primary CD4^+^ T cells. Here, CD45RO^+^ primary T cells proved more sensitive to zanolimumab-induced ADCC than naïve CD45RA^+^T cells.This correlated well with the *in vivo* findings in psoriasis patients, where subcutaneous infusions (once weekly for 4 weeks) resulted in a dose-dependent decrease in the total lymphocyte counts, mainly due to a reduction in CD4^+^ T cells in the memory cell subset (CD3^+^, CD4^+^, CD45RO^+^) [21].

Zanolimumab also effectively induced CD4 down-modulation. This mechanism was found to require CD4 clustering, and to be dependent on the antibodies’ Fc region: whole antibody, but not F(ab')2 fragments, mediated a dose-dependent CD4 down-regulation in the presence of monocytes.

Hence, zanolimumab exerts its action through inhibition of CD4^+^ T cell signaling in concert with the induction of Fc-dependent ADCC and CD4 down-modulation (Fig. 2). This mechanism of action profile, challenging CD4^+^ cells from three different angles, was recognized as being ideal for use in a setting where malignant CD4^+^ T cells pose a threat to patient survival. Such conditions are found in cutaneous T cell lymphoma (CTCL) as well as non-cutaneous T cell lymphoma. CTCL covers a range of diseases, including mycosis fun-goides (MF) and Sézary syndrome, characterized by infiltration of malignant T cells that express CD4 into the skin. The disease is incurable except at a very early stage and is life threatening in the advanced stages. Histologically MF is characterized by the presence of Pautrier microabsesses, in which malignant T cells accumulate in close proximity to APCs [24–26]. It is believed that this close interaction results in chronic stimulation and growth of malignant cells.

**Figure 3:**
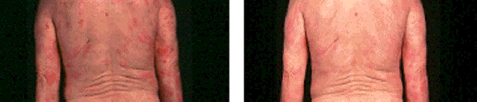
Clinical improvement of mycosis fun-goides. Left panel shows baseline, right panel shows clinical response 4 weeks after start ofzanolimumab treatment. Adapted from [11].

Phase II studies in early and late stage CTCL showed that zanolimumab induced a marked clinical effect, with early, high and durable responses [11] (summarized in Table 2; Fig. 3). At the high dose levels, ten MF patients had objective responses lasting between 8 and 91 weeks, with median response durations of 81 weeks (20.3 months). A Phase III pivotal trial is currently ongoing (Table 3), of which the first interim results indicates a 42% objective response rate in the two highest dose groups [27].

**Table 2:** Key results of clinical studies of zanolimumab, ofatumumab and zalutumumab

Antibody/indication	Publication	Trial description	Dose(s)	Key results

Zanolimumab				
Cutaneous T-cell lymphoma (CTCL)	Kim YH *et al.* [11]	Two Phase II, multi-center, prospective, open-label, uncontrolled trials to evaluate efficacy and safety in patients with treatment refractory CD4^+^ CTCL.	Seventeen weekly i.v. infusion of 280, 560, or 980 mg zanolimumab.	Forty-seven patients were included in the trial. In the high-dose groups (560 and 980 mg), an objective response rate of 56% was obtained in mycosis fungoides patients with a median response of 81 weeks. Adverse effects (AEs) reported most frequently included low-grade infections and eczematous dermatitis.

Non-cutaneous T cell lymphoma	D'Amore F *et al.* [70] (abstract)	A open-label, exploratory trial to explore the efficacy in patients with biopsy proven CD4^+^ peripheral T cell lymphoma of non-cutaneous type who were treatment-refractory or had relapsed.	Twelve weekly i.v. infusions of 980 mg zanolinumab.	Twenty-one patients were included in the trial. Objective tumor response was obtained in 5 out of 21 patients (24%). AEs reported most frequently were rash not otherwise specified and pyrexia. No infections considered related to treatment were reported.

**Ofatumumab**				

Chronic lymphocytic leukemia (CLL)	Coiffier *et al.* [13]	A Phase I/II multicenter, open-label, dose-escalating trial in patients with relapsed or refractory CLL.	Four weekly i.v. infusions of 1 × lOO mg/3×300 mg (cohort A), 1×300 mg/3×1000mg (cohort B), or 1×500 mg/ 3×1000 mg (cohort C).	Thirty-three patients were in cluded in the trial, 3 in cohort A and B, and 27 in cohort C. The maximum tolerated dose (MTD) was not reached. The majority of related AEs occurred at first infusion. Seventeen (51%) patients experienced infections, 88% of them of grade 1–2. One event of interstitial pneumonia was fatal. The response rate of cohort C was 50%.

Follicular lymphoma (FL)	Hagenbeek A *et al.* [71]	A Phase I/II open-label, multicenter trial evaluating safety, efficacy, and pharmacokinetics (PK) in patients with relapsed or refractory FL grade 1–2.	Four weekly i.v. infusions of 300, 500, 700, or 1000 mg in a dose-escalating manner.	Forty patients were included in the trial. MTD was not reached. The majority of related AEs occurred at first infusion and was of grade 1–2. Eight related events were of grade 3. The best objective clinical response across dose groups was 43%. Median time to progression for all patients/responders was 8.8/32.6 months and median duration of response was 29.9 months at a median/maximum follow-up of 9.2/38.6 months. The median half-life across dose groups was 410 h after the fourth dose.
Squamous cell carcinoma of the head and neck (SCCHN)	Bastholt L *et al.* [64]	A Phase I/II open-label, multicenter trial evaluating safety, tolerability, PK, and efficacy in patients with SCCHN	A single i.v. infusion of HuMax-EGFR at doses of 0.15, 0.5, 1, 2, 4, or 8 mg/kg i.v. (dose-escalating) followed by four weekly i.v. infusions at the same doses (repeat dose extension).	Twenty-eight patients were included in the trial. MTD was not reached. The most frequently reported AE was rash (duration: a few days to 2 months). All but one event were of grade 1–2 and a dose-dependent relationship was indicated. In the two highest dose groups, 7 of 11 patients obtained a partial response (PR) or stable disease (SD) and 9 patients obtained metabolic PR or SD.

Overall, our approach shows that CD4 targeting can be extended well beyond (auto)immune indications. By combining effective engagement of ADCC with direct effects on CD4 expression and signaling, we generated a promising new antibody drug for the treatment of for CD4^+^ cell malignancies. We are currently not only developing this antibody for treatment of CTCL, but are also exploring its potential in peripheral T cell lymphomas (Table 3).

**Table 3:** Clinical development in cancer indications of zanolimumab, ofatumumab and zalutumumaba)

Zanolimumab		
*Previous studies*		
Zanolimumab in early stage CTCL	CTCL	Phase II
Zanolimumab in late stage CTCL	CTCL	Phase II
Zanolimumab in refractory and relapsed non-CTCL	Non-CTCL	Phase II
*Ongoing studies*		
Zanolimumab in combination with CHOP chemotherapy in non-CTCL	Non-CTCL	Phase II combination study
Zanolimumab in CTCL refractory to standard therapy	CTCL	Phase III pivotal study

**Ofatumumab**b)		

*Previous studies*		
Ofatumumab in relapsed or refractory follicular NHL	Follicular NHL	Phase I/II
Ofatumumab in relapsed CLL	CLL	Phase I/II
*Ongoing studies*		
Ofatumumab in follicular NHL refractory to rituximab therapies	Follicular NHL	Phase III pivotal study
Ofatumumab in combination with CHOP	Follicular NHL	Phase II combination study
Ofatumumab in refractory CLL	CLL	Phase III pivotal study
Ofatumumab in combination with fludarabine and cyclophosphamide	CLL	Phase II combination study
Ofatumumab in relapsed DLBCL	DLBCL	Phase II study

**Zalutumumab**		

*Previous studies*		
Zalutumumab in patients with recurrent or metastatic SCCHN	SCCHN	Phase I/II
*Ongoing studies*		
Zalutumumab in combination with chemotherapy and radiotherapy in head and neck cancer	SCCHN	Phase I/II combination study
Zalutumumab in patients with non-curable head and neck cancer	SCCHN	Phase III pivotal study
DAHANCA 19: The importance of the EGFR-inhibitor zalutumumab for the outcome after curative radiotherapy for SCCHN	SCCHN	Phase III study
Zalutumumab in combination with chemo-radiation in lung cancer	NSCLC	Phase I/II combination study
Zalutumumab in non-curable patients with SCCHN	SCCHN	Phase II study
Zalutumumab with or without irinotecan chemotherapy in cetuximab-refractory colorectal cancer	CRC	Phase I/II combination study

a) Status in May 2008. For the most current information on clinical development of Zanolimumab, ofatumumab and zalutumumab, see www.genmab.com and www.clinicaltrials.gov. CTCL, cutaneous T cell lymphoma; CLL, chronic lymphocytic leukemia; NHL, non-Hodgkin's lymphoma; DLBCL, diffuse large B cell lymphoma; SCCHN, squamous cell carcinoma of the head and neck; CRC, colorectal cancer; NSCLC, non-small cell lung cancer

b) For ofatumumab, only studies in cancer indications are listed here. Ofatumumab is also in clinical development in rheumatoid arthritis and multiple sclerosis.

## 4 Ofatumumab: Efficient inducer of CDC through a unique binding epitope

**Figure 4:**
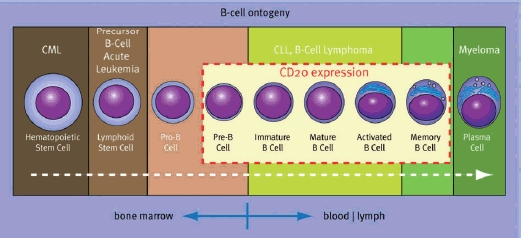
CD20 expression in B cell ontogeny. B cell development is a multi-staged process that begins with a pluripotent hematopoietic stem cell and ends with the formation of an antibody-producing plasma cell. CD20 expression is restricted to the pre-B cell to memory B cell stage. B cell malignancies, as indicated, can occur at almost any stage of B cell development, producing a variety of distinct leukemias and lymphomas.

CD20 is arguably one of the best-validated targets for antibody therapy [28, 29]. On normal cells, CD20 is first expressed at the pre-B cell stage, before IgM is expressed on the cell surface (Fig. 4). The expression of CD20 continues throughout B cell maturation until the plasmacytoid immunoblast phase, yet CD20 is not expressed on lymphoid stem cells or plasma cells. In B cell malignancies, like B cell NHL, CD20 is highly expressed. Besides being an apparent target in oncology, CD20 targeting is now also applied in autoimmune diseases, such as rheumatoid arthritis.

CD20 represents a non-glycosylated, 297-amino acid (33–37 kDa) phosphoprotein that is predicted to span the plasma membrane four times [30]. Both the N and C termini of the protein are located within the cytoplasm. A large loop of 44 amino acids and a much smaller loop of 7 amino acids are accessible from the extracellular milieu.

The function of CD20 is not known. It has been suggested to play a role in B cell activation, the regulation of B cell growth, and transmembrane calcium flux. The structure of CD20 implies that it either forms an ion channel or is associated with an ion channel [31]. Interestingly, upon cross-linking induced by a number of CD20 antibodies, CD20 translocates to cholesterol- and sphingolipid-rich microdomains, so-called ‘lipid rafts’ [32]. A central feature of these rafts is their ability to selectively include or exclude membrane proteins. Translocation into lipid rafts is an extremely rapid process, and is directly followed by phosphorylation of typical raft proteins like the protein tyrosine kinase Lyn, which initiates signaling cascades. This indicates that translocation of CD20 into lipid rafts is necessary to trigger signaling upon CD20 cross-linking.

One of the first chimeric antibodies to enter the clinic, rituximab [brand names Rituxan (USA) and MabThera (Europe)], has validated CD20 as a prominent therapeutic target. Rituximab is now used routinely in the treatment of NHL, either as a single agent or (most often) in combination with chemotherapy. Yet, in other B cell malignancies such as chronic lymphocytic leukemia (CLL), rituximab appears far less effective.

**Figure 5:**
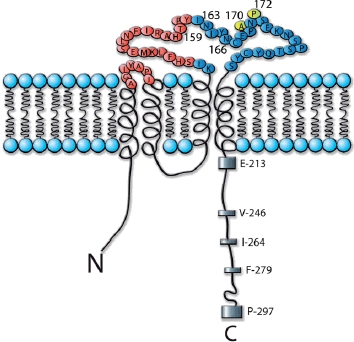
Epitope of ofatumumab. Peptide scanning and mutation studies revealed the binding epitope of ofatumumab on CD20. Amino acids contributing to ofatumumab binding are indicated in red [35]. Amino acids essential for rituximab, but not ofatumumab binding are indicated in yellow [36, 37].

Ofatumumab (clone 2F2, IgG1κ) was generated amongst a panel of fully human CD20 mAbs [33]. Characterization of this panel and comparison to rituximab identified ofatumumab as an extremely potent inducer of CDC, in addition to it being able to effectively induce ADCC (Fig. 2) [33, 34]. The distinction of ofatumumab with regard to CDC induction was found to be linked to binding to a unique epitope on CD20 [35]. Epitope mapping studies using a mutagenesis approach revealed that, whereas the epitope for rituximab lies entirely within the larger of the two extracellular loops [36, 37], ofatumumab binds to a motif that includes the small extracellular loop (Fig. 5) [35]. With this small loop being located close to the cell membrane, it is hypothesized that ofatumumab binding allows a very efficient localization of complement on the cell surface. Indeed, increased C1q binding, leading to more C4c and C3b fixation, and enhanced CDC activity have been observed with ofatumumab compared to rituximab [33, 34, 38]. For instance, Bmax for binding of C4c to ofatumumab-coated Daudi cells is 3507 (mean fluorescence intensity, measured in FACS analysis) *versus* 2410 for rituximab, with corresponding EC_50_ values of 0.05 μg/mL *versus* 0.42μg/mL [33].

**Figure 6:**
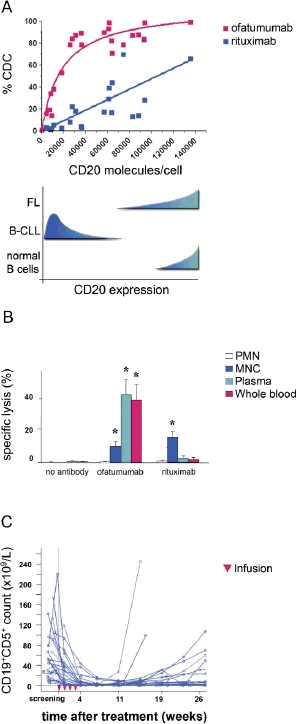
Potent B cell killing capacity of ofatumumab. (A) Anti-CD20-mediated CDC of CEM cells expressing varying amounts of CD20. The range of CD20 expression in follicular lymphoma and B cell chronic lymphatic leukemia is indicated, showing the potential for ofatumumab of killing cells that are resistant to rituximab. (B) Primary CLL cells are efficiently killed by ofatumumab with human effectors. Human blood (whole blood) was fractionated into polymorphonuclear (PMN) or mononuclear cells (MNC), or into complement containing plasma. Specific target cell lysis was assessed in ^51^Cr release assays (*, significant difference compared to “no antibody", p < 0.001). (C) Leukemic B cell counts in B-CLL patients receiving four weekly infusions (1×500 mg and 3×1000 mg) of ofatumumab. Adapted from [13, 33, 35].

Ofatumumab not only induced CDC at low antibody concentrations, it was also able to lyse cells with low levels of CD20 expression and those with high expression of CDC-defense molecules CD55 and CD59. Using a panel of CD20-transduced human CEM T cell clones expressing varying numbers of CD20 molecules, ofatumumab was found to lyse cells with as few as 4000 CD20 molecules at their cell surface (Fig. 6A). CD20-low-expressing, rituximab-resistant CLL cells were also efficiently lysed by ofatumumab (Fig. 6B) [33]. Pharmacokinetic analyses in SCID mice (in which no antibody response against the injected antibody can be mounted) indicated expected serum half-life times *in vivo*: 19 ± 0.4 days for ofatumumab, 15 ± 0.8 days for rituximab^2^.

The pharmacological results for ofatumumab indicated its clinical potential: being an effective killer of (malignant) B cells, ofatumumab could be efficacious as monotherapy in addition to having an expected enhanced efficacy in combination with other agents. Patients with an existing or acquired resistance to other (CD20 directed) therapies might benefit from a more potent ofatumumab-based therapy. Interestingly, acquired resistance to rituximab was recently found to be associated with down-regulation of CD20 expression and up-regulated expression levels of complement-regulatory protein CD55 [39]. In addition, B cell malignancies characterized by low CD20 expression levels, such as CLL, or those with high expression levels of complement-regulatory proteins may now be eligible for CD20 directed therapy with ofatumumab.

Results from Phase I/II studies in B-CLL and follicular lymphoma supported the therapeutic potential of ofatumumab. Ofatumumab safety and efficacy was tested in a multicenter, dose-escalating study of 33 patients with relapsed or refractory CLL [13]. The results of this study are summarized in Table 2. Following four weekly ofatumumab doses, a rapid, substantial and consistent B cell depletion was observed (Fig. 6C), and a 50% overall response rate reached. Importantly, ofatumumab was well tolerated, and the maximum tolerated dose was not reached. The primary safety observation was occurrence of infusion reactions in patients receiving the highest dose; however, their occurrence decreased with subsequent dosing and the incidence was comparable to that described for rituximab. Hence, the potent CDC induction by ofatumumab did not appear to trigger increased systemic adverse reactions. Again, the close binding proximity of ofatumumab to the cell membrane likely results in highly efficient complement deposition on B cell membranes, without high levels of systemic release of activated complement components.

In follicular NHL, a Phase I/II dose-escalation trial included 40 patients receiving four weekly ofatumumab infusions (Table 2). Again, a rapid and sustained B cell depletion was observed at all doses, with B cell recovery corresponding to dose levels, and an overall response rate up to 63% was reached. Notably, in a subgroup of patients who previously received rituximab therapy but had relapsed, the overall response rate was 64%. In contrast, re-treatment with rituximab has been reported to result in an overall response rate of 35–40% [40, 41].

In both CLL and follicular NHL, ofatumumab is now being evaluated in pivotal Phase III studies. In addition, a Phase II study in relapsed diffuse large B cell lymphoma patients has been initiated. For all these B cell malignancies, it is understood that there is clear potential for CD20-directed therapy (either as monotherapy or in combination with other treatment modalities). With ofatumumab having such a strong CDC activity, in addition to potent ADCC, even on cells with high expression of complement regulatory proteins or low expression of CD20, it can be expected that this new human antibody will be able to make a difference for patients resistant or refractory to current therapeutics.

## 5 Zalutumumab: Disconnecting EGFR molecules, connecting effector cells

The central role of the EGFR/ErbB family members in cell migration, proliferation, survival, and transformation identified these transmembrane receptors as targets for cancer therapeutic approaches. This family includes ErbB1/HER1/EGFR, ErbB2/HER2/Neu, ErbB3/HER3 and ErbB4/HER4 [42]. The first ErbB receptor-targeting antibody approved by the United States FDA in 1998 was trastuzumab (brand name Herceptin), for the treatment of HER2-positive breast cancer. For targeting of EGFR/HER1, cetuximab (brand name Er-bitux) and panitumumab (brand name Vectibix) are currently approved for clinical use.

Zalutumumab (clone 2F8) targets EGFR, a 170-kDa transmembrane glycoprotein containing a tyrosine kinase domain and regulatory domain in the C terminus [43]. EGFR is expressed on normal cells at levels ranging from 20 000 to 200 000 receptors per cell. The intracellular kinase domain is activated upon ligand binding to the EGFR extracellular domain. EGFR plays a role during embryogenesis in the morphogenesis of organs like teeth, brain, reproductive and gastrointestinal tracts, and cardiovascular system. Physiologically, EGFR has an essential role in wound healing and normal epithelial regeneration of organs. EGFR activation has been described to trigger processes such as proliferation, apoptosis, migration, angiogenesis and differentiation [44–47].

Rowinsky *et al.* [48] showed that EGFR plays a role in the transformation and progression of carcinomas. EGFR overexpression in tumor cells can produce up to 2 million EGFR molecules per cell, and has been correlated to a more malignant tumor grade as well as a reduced patient survival [48, 49]. Additionally, mutant forms of EGFR and overexpression of EGFR ligands have been recognized to promote cancer progression in a range of solid tumors.

Next to mAbs, another class of drugs is successfully employed to target EGFR for cancer therapy: small molecule tyrosine kinase inhibitors (TKI), targeting the ATP-binding pocket of the EGFR kinase domain. TKI, including the U.S. FDA and European Medicine Agency (EMEA) approved agents gefitinib (Iressa), erlotinib (Tarceva) and lapatinib (Tykerb), are oral, low-molecular-weight inhibitors of the EGFR tyrosine kinase located in the intracellular part of the receptor. TKI block EGFR kinase activity and EGFR downstream signaling [50]. In contrast to the approach using mAb, tyrosine kinase inhibition is not strictly EGFR-specific and some cross-reactivity with other ErbB family members, *e.g.*, HER2, may occur [51]. mAbs and TKI also differ in pharmacological and pharmacokinetic properties. EGFR antibodies, being large proteins (∼150 kDa), are normally administered intravenously in contrast to TKI, which are small synthetic molecules (∼500 Da) and are taken orally. According to the FDA labels, antibody half-lives in blood (for cetuximab 3.1–7.8 days; for panitumumab 3.6–11.9 days, allowing for dosing every 7, or 14 days, respectively) are longer than those of small-molecule agents (gefitinib, ∼48 h; erlotinib, ∼36 h; allowing for once-daily dosing). Further, pharmacokinetic studies demonstrated that plasma concentrations of TKI vary substantially between patients [52].

An important functional difference between TKI and EGFR antibodies lies in the antibodies' ability to attack cancer cells through a combination of mechanisms. TKI, being small, are indeed able to pass through cell membranes and can act on targets in the cytoplasm regardless of their cellular location, yet once in the cell they do only one thing: inhibit kinase activity. For several types of cancer, certain (secondary) mutations in the EGFR kinase domain have been reported that confer resistance to TKI [53–55]. For antibodies, being able to recruit immune effector functions in addition to having direct effects on EGFR activity, resistance to therapy is unlikely to arise through mutations in the EGFR kinase domain. Indeed (as described below), clinically relevant EGFR mutations did not influence the activity of EGFR antibodies.

Zalutumumab, a high-affinity human IgG1K EGFR antibody, potently inhibits tumor growth in xenograft models by engaging two mechanisms of action (Fig. 2) [56]. Firstly, EGFR signaling is blocked. This was observed as a reduction in receptor phosphorylation and is most effective at saturating antibody concentrations. Secondly, anti-tumor effects are mediated by Fc-mediated ADCC. This mechanism is already active at low target occupancy. Together, these properties make zalutumumab highly effective in cancer disease models, being able to eradicate tumors even at low and infrequent doses.

As inhibition of EGFR signaling was recognized as an important mechanism not only of zalutumumab, but also of other EGFR antibodies like cetuximab, we investigated the influence of (lung cancer-derived) mutations that change the EGFR kinase activity, on tumor cell killing [57] .An EGFR-responsive cell line model was established, in which cells expressed tumor-derived EGFR mutations (L858R, G719S, delE746-A750 and secondary mutation T790M). In these, two prototypic TKI, gefitinib and erlotinib, demonstrated the expected reduced or abolished activities. In sharp contrast, anti-tumor effects of zalutumumab and cetuximab were not impacted by these mutations. Not only did inhibition of EGFR signaling and cell growth by the EGFR antibodies remain intact, their ADCC activity was also unaffected.

**Figure 7:**
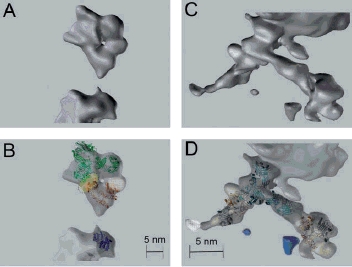
Conformation of zalutumumab-bound EGFR. Shown are tomograms of zalutumumab-bound EGFR. In the lower panels, the tethered crystal structure of sEGFR (PDB: 1 nql, shown as a ribbon representation) was superimposed into EGFR ectodomain tomograms. The crystal structure of human immunoglobulin 1 ﹛PDB: (A) 1 HZH [57], (B) 1 IGY [58]﹜ was superimposed into zalutumumab (green). Panels A and B show a tomogram of a zalutumumab molecule monovalently bound to EGFR. The complex was marked by anti-EGFR-3.5-nm colloidal gold-conjugated protein A intracellular labeling only. Dotted line in (B) marks the zalutumumab docking site on EGFR. The EGFR ectodomain structure is condensed and resembles the tethered EGFR conformation, when zalutumumab is bound (*n*=4). (C, D) Tomograms in which one zalutumumab antibody molecule binds two EGFR molecules. Zalutumumab binds one EGFR molecule with each of its Fab arms, spatially separating the two receptors (*n*=2). The extra volume present on EGFR domain I (white) likely represents carbohydrate chains. From [58].

Remarkably, it was found that, while zalutumumab efficiently inhibited EGFR signaling, the intact antibody was more efficient in this than Fab fragments [58]. Hence, bivalent binding to EGFR contributed to zalutumumab's mechanism of action. To understand the molecular mechanisms underlying this, we used protein tomography to visualize EGFR conformations on EGFR-overexpress-ing cells in its monomeric (resting), its EGF-stimu-lated conformation and its zalutumumab-inhibited conformation [58] (Fig. 7). This technique allowed us to observe individual cell membrane-localized EGFR molecules at a level of detail not previously obtainable, *i.e.*, where separate protein domains could be discerned. Monomeric, resting EGFR ectodomains were found to be highly flexible molecules, showing that EGFR is able to switch between a tethered (auto-inhibited) conformation and extended (pre-activated) “pistol-shaped” conformation (as described before, [59, 60]). In contrast, zalutumumab-bound EGFR conformations were condensed and appeared to be locked in one specific, very compact (and inactive) conformation. Bivalent binding and EGFR cross-linking by zalutumumab was found to spatially separate the EGFR molecules’ intracellular kinase domains to an extent, which appeared incompatible with kinase domain interaction and induction of signaling.

For other EGFR antibodies (in particular for cetuximab), antibody-induced down-modulation of EGFR has been postulated as part of the mechanism of action [61, 62]. Zalutumumab was found to induce moderate EGFR down-modulation [63].

**Figure 8:**
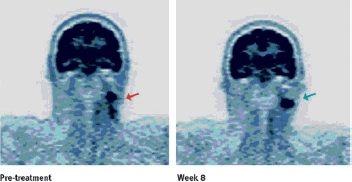
Clinical effect of zalutumumab. PET scan from a patient with SCCHN showing a partial metabolic response upon treatment with zalutumumab (8 mg/kg).

Having potent anti-tumor activity in laboratory settings, even at doses where other EGFR antibodies were found to be much less effective, zalutumumab was recognized as having the properties required for treatment of a variety of cancers, and was awarded fast track status from the U.S. FDA for head and neck cancer patients who have failed standard therapies (Table 3). Its efficacy is also explored for the treatment of non-small cell lung cancer, in combination with chemo-radiation therapy, and colorectal cancer in combination with irinotecan chemotherapy. Phase I/II results in squamous cell carcinoma of the head and neck (SCCHN) showed good responses, as demonstrated by two types of scanning (Table 2) [64] .Assessed by FDG-PET, which visualizes tumor metabolism, 7 of 18 evaluable patients achieved partial metabolic response (PMR) (Fig. 8) and 4 had stable metabolic disease (SMD) 1 week after their fifth and last infusion. In the two highest dose groups, 9 out of 11 patients obtained PMR or SMD. These results were confirmed by computerized tomography (CT scan).

At present, the field of EGFR-targeted therapeutics is evolving rapidly as better insights in EGFR biology and clinical trial data become available. Although objective clinical responses have been frequently observed during EGFR-targeted therapies, many patients develop therapy resistance. Here, it becomes clear that a better understanding of the mechanism of action of EGFR therapeutics, and studies on how tumor cell resistance can be circumvented will further contribute to optimize treatment regimes. Indeed, zalutumumab, displaying novel modes of action that distinguish it from other EGFR antibodies, has potential as effective treatment in a variety of cancers.

## 6 Umabs: The new standard

Antibody therapeutics make a profound impact on the treatment of cancer. The technologies used to generate new therapeutic antibodies have become highly refined. The first -omabs brought promise but also disappointments, and it was the -ximabs and -zumabs that set the stage for antibody therapy. Now, -umabs are becoming the standard.

These fully human antibodies can be generated using various techniques, including phage display and transgenic technologies. The characteristics of antibodies generated from these platforms are intrinsically determined by the human V repertoire contained in the libraries/transgenic mice, and the extent of (*in vitro* or *in vivo*) affinity maturation that occurs. Overall, these determine the pool of antibodies from which potential development candidates can be selected. The quality of the final clinical candidate, however, is strongly dependent on the entire lead candidate selection process. We believe that our approach of antibody library generation followed by rational and science-based library screening, significantly contributes to selecting an optimal final product.

Several other methods of improving the response rates of mAbs in cancer therapy have been investigated and are now in early clinical evaluation. For instance, combinations of antibodies are being explored as an approach to overcome the often-observed tumor escape from antibody therapy. An example here is the combination of HER2 mAbs recognizing different epitopes on the HER2 extracellular domain, which were found to give superior anti-tumor activity compared to the individual antibodies, both *in vitro* and *in vivo* [65]. Also, the feasibility of combining various antibodies with ritux-imab, *e.g.,* Campath, has been investigated [66]. Recently, we have reported a remarkable synergy for EGFR antibodies [67]. Individually, these antibodies have potent activity in ADCC (as described above), whereas complement-mediated lysis is not apparent. Combining IgG1 EGFR-antibodies recognizing different, non-overlapping epitopes, however, resulted in potent complement activation and effective lysis of tumor cells. These findings indicate unexpected qualities of EGFR antibody combinations that can be deployed for novel cancer treatments.

It is recognized that certain therapeutic applications may benefit from optimizations of the antibody format, and this is sprouting an extensive line of research focusing on engineering for enhanced effector function, controlling of half-life, increasing stability and improving tumor and tissue penetration (extensively reviewed in [68]). For instance, the affinity of the Fc domain to Fc receptors can be modified by changing the sugar structures on antibodies (glyco-engineering) [69]. With this, efficacies can be increased, dosing lowered and potentially side effects minimized.

The continuing increase in knowledge of antibody biology and mechanisms of action, and the application of this knowledge in understanding the antibodies interactions at molecular, cellular and organism levels, will enable us to further improve and expand the therapeutic application of antibodies in human diseases. Zanolimumab, ofatumumab and zalutumumab illustrate how each therapeutic antibody harbors unique features, which can be exploited to optimally target specific cancer indications, and provide new treatment options for patients.

**Sigrid Ruuls** holds a PhD in immunology from the Free University of Amsterdam, where she studied the role of macrophages in multiple sclerosis and its animal model counterpart, experimental allergic encephalomyelitis. She worked as a post doctoral researcher in the group of Dr. Jonathon Sedgwick at the Centenary Institute in Sydney, Australia and DNAX, Palo Alto, USA. Here she investigated the role of membrane-bound TNF in secondary lymphoid organ formation and the pathogenesis of autoimmune inflammation. Since 2001, she is Scientific Communication Manager at Genmab in Utrecht, the Netherlands, responsible for regulatory and scientific documentation supporting the research and development of human antibody therapeutics.

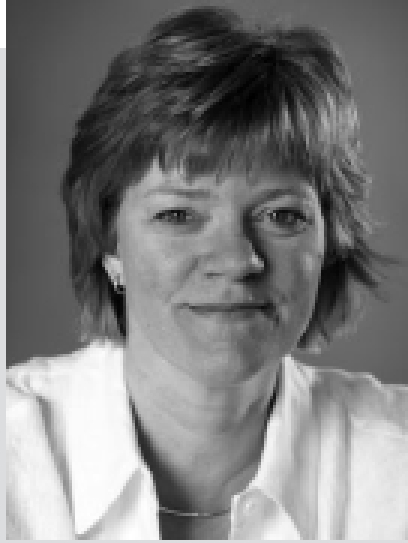

**Paul Parren** holds a PhD in molecular immunology from the University of Amsterdam, where he studied effector functions of recombinant antibodies. He was an Associate Professor at The Scripps Research Institute in La Jolla, California. Here he unraveled mechanisms of antibody-mediated protection against viral infections. Since 2002, he has been at Genmab in Utrecht, the Netherlands, where he serves as Sr. Vice President, Research and Pre-Clinical development and works on the research and development of human antibody therapeutics. He is dedicated to advancing the field of therapeutic antibodies by the science-based development of novel and/or improved antibody-based therapies for combating human disease.

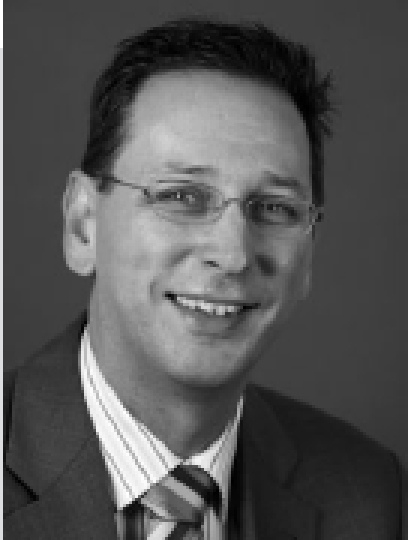

## Footnotes

1 The anti-antibody responses in humans are abbreviated according to the type of molecule they are directed against: HAMA for human anti-murine antibodies, HACA for human anti-chimeric antibodies and HAHA for human anti-humanized or human anti-human antibodies.

2 In addition to rituximab, two other CD20-antibodies have been FDA-approved for human use: Bexxar (tositumomab and ^131^I-labeled tositu-momab) and Zevalin (^90^Y- or 111 In-labeled ibritumomab tiuxetan). These antibodies are used in a different therapeutic setting, namely as radioim-munotherapeutic, which benefits from the relatively short half-life of mouse antibodies in humans. Whereas Zevalin is based on the same mouse antibody parent as rituximab (*i.e.*, C2B6), Bexxar is based on B1, an antibody with an intrinsic activity that is entirely different from ofatumumab and rituximab/C2B6 (reviewed in [29]). It is relatively inactive in complement activation, but in contrast promotes programmed cell death.

## Abbreviations

ADCC: antibody-dependent cellular cytotoxicity
APC: antigen-presenting cell
CDC: complement-dependent cytotoxicity
CDR: complementarity determining region
CLL: chronic lymphocytic leukemia
EGFR: epidermal growth factor receptor
FDA: Food and Drug Administration
HLA: human leukocyte antigens
MHC: major histocompatibility complex
NHL: non-Hodgkin's lymphoma
SCCHN: squamous cell carcinoma of the head and neck
TKI: tyrosine kinase inhibitor

## References

[1] Ortho Multicenter Transplant Study Group (1985). A randomized clinical trial of OKT3 monoclonal antibody for acute rejection of cadaveric renal transplants. N. Engl. J. Med..

[2] Morrison SL, Johnson MJ, Herzenberg LA, Oi VT (1984). Chimeric human antibody molecules: Mouse antigen-binding domains with human constant region domains. Proc. Natl. Acad. Sci. USA.

[3] Jones PT, Dear PH, Foote J, Neuberger MS (1986). Replacing the complementarity-determining regions in a human antibody with those from a mouse. Nature.

[4] Lonberg N (2005). Human antibodies from transgenic animals. Nat. Biotechnol..

[5] Fishwild DM, O'Donnell SL, Bengoechea T, Hudson DV (1996). High-avidity human IgG kappa monoclonal antibodies from a novel strain of minilocus transgenic mice. Nat. Biotechnol..

[6] Lonberg N (2008). Fully human antibodies from transgenic mouse and phage display platforms. Curr. Opin. Immunol..

[7] Schellekens H (2002). Bioequivalence and the immunogenicity of biopharmaceuticals. Nat. Rev. Drug Discov..

[8] Van Walle I, Gansemans Y, Parren PW, Stas P (2007). Immunogenicity screening in protein drug development. Expert Opin. Biol. Ther..

[9] Jakobovits A, Amado RG, Yang X, Roskos L (2007). From XenoMouse technology to panitumumab, the first fully human antibody product from transgenic mice. Nat. Biotechnol..

[10] Kellermann SA, Green LL (2002). Antibody discovery: The use of transgenic mice to generate human monoclonal antibodies for therapeutics. Curr. Opin. Biotechnol..

[11] Kim YH, Duvic M, Obitz E, Gniadecki R (2007). Clinical efficacy of zanolimumab (HuMax-CD4): Two phase 2 studies in refractory cutaneous T-cell lymphoma. Blood.

[12] Knox SJ, Levy R, Hodgkinson S, Bell R (1991). Observations on the effect of chimeric anti-CD4 monoclonal antibody in patients with mycosis fungoides. Blood.

[13] Coiffier B, Lepretre S, Pedersen LM, Gadeberg O (2008). Safety and efficacy of ofatumumab, a fully human monoclonal anti-CD20 antibody, in patients with relapsed or refractory B-cell chronic lymphocytic leukemia: A phase 1-2 study. Blood.

[14] Magdelaine-Beuzelin C, Kaas Q, Wehbi V, Ohresser M (2007). Structure-function relationships of the variable domains of monoclonal antibodies approved for cancer treatment. Crit. Rev. Oncol. Hematol..

[15] Harding S, Lipp P, Alexander DR (2002). A therapeutic CD4 monoclonal antibody inhibits TCR-zeta chain phosphorylation, zeta-associated protein of 70-kDa Tyr319 phosphorylation, and TCR internalization in primary human T cells. J. Immunol..

[16] Marschner S, Hunig T, Cambier JC, Finkel TH (2002). Ligation of human CD4 interferes with antigen-induced activation of primary T cells. Immunol. Lett..

[17] Herzog C, Walker C, Muller W, Rieber P (1989). Anti-CD4 antibody treatment of patients with rheumatoid arthritis: I. Effect on clinical course and circulating T cells. J. Autoimmun..

[18] Choy EH, Connolly DJ, Rapson N, Jeal S (2000). Pharmacokinetic, pharmacodynamic and clinical effects of a humanized IgG1 anti-CD4 monoclonal antibody in the peripheral blood and synovial fluid of rheumatoid arthritis patients. Rheumatology (Oxford).

[19] Winsor-Hines D, Merrill C, O'Mahony M, Rao PE (2004). Induction of immunological tolerance/hyporesponsiveness in baboons with a nondepleting CD4 antibody. J. Immunol..

[20] Reimann KA, Khunkhun R, Lin W, Gordon W (2002). A humanized, nondepleting anti-CD4 antibody that blocks virus entry inhibits virus replication in rhesus monkeys chronically infected with simian immunodeficiency virus. AIDS Res. Hum. Retroviruses.

[21] Skov L, Kragballe K, Zachariae C, Obitz ER (2003). Hu-Max-CD4: A fully human monoclonal anti-CD4 antibody for the treatment of psoriasis vulgaris. Arch. Dermatol..

[22] Rider DA, Havenith CE, de Ridder R, Schuurman J (2007). A human CD4 monoclonal antibody for the treatment of T-cell lymphoma combines inhibition of T-cell signaling by a dual mechanism with potent Fc-dependent effector activity. Cancer Res..

[23] Fishwild DM, Hudson DV, Deshpande U, Kung AH (1999). Differential effects of administration of a human anti-CD4 monoclonal antibody, HM6G, in nonhuman primates. Clin. Immunol..

[24] Hansen ER, Baadsgaard O, Lisby S, Cooper KD (1990). Cutaneous T-cell lymphoma lesional epidermal cells activate autologous CD4^+^ T lymphocytes: Involvement of both CD1^+^OKM5^+^ and CD1^+^OKM5^−^ antigen-presenting cells. J. Invest. Dermatol..

[25] Lisby S, Baadsgaard O, Cooper KD, Thomsen K (1988). Expression of OKM5 antigen on epidermal cells in mycosis fungoides plaque stage. J. Invest. Dermatol..

[26] Lisby S, Baadsgaard O, Cooper KD, Hansen ER (1990). Phenotype, ultrastructure, and function of CD1^+^DR^+^ epidermal cells that express CD36 (OKM5) in cutaneous T-cell lymphoma. Scand. J. Immunol..

[27] Duvic M, Kim YH, Korman NJ, Bogh E (2006). Zanolimumab, a fully human monoclonal antibody: Early results of an ongoing clinical trial in patients with CD4^+^ mycosis fungoides-type cutaneous T-cell lymphoma (stage IB–IVB) who are refractory or intolerant to Targretin(r) and one other standard.

[28] Martin F, Chan AC (2006). B cell immunobiology in disease: Evolving concepts from the clinic. Annu. Rev. Immunol..

[29] Glennie MJ, French RR, Cragg MS, Taylor RP (2007). Mechanisms of killing by anti-CD20 monoclonal antibodies. Mol. Immunol..

[30] Riley JK, Sliwkowski MX (2000). CD20: A gene in search of a function. Semin. Oncol..

[31] Li H, Ayer LM, Lytton J, Deans JP (2003). Store-operated cation entry mediated by CD20 in membrane rafts. J. Biol. Chem..

[32] Deans JP, Robbins SM, Polyak MJ, Savage JA (1998). Rapid redistribution of CD20 to a low density detergent-insoluble membrane compartment. J. Biol. Chem..

[33] Teeling JL, French RR, Cragg MS, van den Brakel J (2004). Characterization of new human CD20 monoclonal antibodies with potent cytolytic activity against non-Hodgkin lymphomas. Blood.

[34] Bleeker WK, Munk ME, Mackus WJ, van den Brakel JH (2007). Estimation of dose requirements for sustained *in vivo* activity of a therapeutic human anti-CD20 antibody. Br. J. Haematol..

[35] Teeling JL, Mackus WJ, Wiegman LJ, van den Brakel JH (2006). The biological activity of human CD20 monoclonal antibodies is linked to unique epitopes on CD20. J. Immunol..

[36] Polyak MJ, Deans JP (2002). Alanine-170 and proline-172 are critical determinants for extracellular CD20 epitopes; heterogeneity in the fine specificity of CD20 monoclonal antibodies is defined by additional requirements imposed by both amino acid sequence and quaternary structure. Blood.

[37] Perosa F, Favoino E, Caragnano MA, Dammacco F (2006). Generation of biologically active linear and cyclic peptides has revealed a unique fine specificity of rituximab and its possible cross-reactivity with acid sphingomyelinase-like phosphodiesterase 3b precursor. Blood.

[38] Beum PV, Lindorfer MA, Beurskens F, Stukenberg PT (2008). Complement activation on B lymphocytes opsonized with rituximab and ofatumumab produces substantial changes in membrane structure preceding cell lysis. J. Immunol..

[39] Czuczman MS, Olejniczak S, Gowda A, Kotowski A (2008). Acquirement of rituximab resistance in lymphoma cell lines is associated with both global CD20 gene and protein down-regulation regulated at the pretranscriptional and posttranscriptional levels. Clin. Cancer Res..

[40] Davis TA, Grillo-Lopez AJ, White CA, McLaughlin P (2000). Rituximab anti-CD20 monoclonal antibody therapy in non-Hodgkin's lymphoma: Safety and efficacy of re-treatment. J. Clin. Oncol..

[41] Hainsworth JD, Litchy S, Shaffer DW, Lackey VL (2005). Maximizing therapeutic benefit of rituximab: Maintenance therapy *versus* re-treatment at progression in patients with indolent non-Hodgkin's lymphoma – A randomized phase II trial of the Minnie Pearl Cancer Research Network. J. Clin. Oncol..

[42] Salomon DS, Brandt R, Ciardiello F, Normanno N (1995). Epidermal growth factor-related peptides and their receptors in human malignancies. Crit. Rev. Oncol. Hematol..

[43] Ullrich A, Coussens L, Hayflick JS, Dull TJ (1984). Human epidermal growth factor receptor cDNA sequence and aberrant expression of the amplified gene in A431 epidermoid carcinoma cells. Nature.

[44] Carpenter G, Cohen S (1990). Epidermal growth factor. J. Biol. Chem..

[45] Cao L, Yao Y, Lee V, Kiani C (2000). Epidermal growth factor induces cell cycle arrest and apoptosis of squamous carcinoma cells through reduction of cell adhesion. J. Cell Biochem..

[46] Armstrong DK, Kaufmann SH, Ottaviano YL, Furuya Y (1994). Epidermal growth factor-mediated apoptosis of MDA-MB-468 human breast cancer cells. Cancer Res..

[47] Yarden Y (2001). The EGFR family and its ligands in human cancer. signalling mechanisms and therapeutic opportunities. Eur. J. Cancer.

[48] Rowinsky EK (2004). The erbB family: Targets for therapeutic development against cancer and therapeutic strategies using monoclonal antibodies and tyrosine kinase inhibitors. Annu. Rev. Med..

[49] Gullick WJ (1991). Prevalence of aberrant expression of the epidermal growth factor receptor in human cancers. Br. Med. Bull..

[50] Imai K, Takaoka A (2006). Comparing antibody and small-molecule therapies for cancer. Nat. Rev. Cancer.

[51] Arteaga CL (2001). The epidermal growth factor receptor: From mutant oncogene in nonhuman cancers to therapeutic target in human neoplasia. J. Clin. Oncol..

[52] Dancey J, Sausville EA (2003). Issues and progress with protein kinase inhibitors for cancer treatment. Nat. Rev. Drug Discov..

[53] Pao W, Miller VA, Politi KA, Riely GJ (2005). Acquired resistance of lung adenocarcinomas to gefitinib or erlotinib is associated with a second mutation in the EGFR kinase domain. PLoS Med..

[54] Kosaka T, Yatabe Y, Endoh H, Yoshida K (2006). Analysis of epidermal growth factor receptor gene mutation in patients with non-small cell lung cancer and acquired resistance to gefitinib. Clin. Cancer Res..

[55] Kobayashi S, Boggon TJ, Dayaram T, Janne PA (2005). EGFR mutation and resistance of non-small-cell lung cancer to gefitinib. N. Engl. J. Med..

[56] Bleeker WK, Lammerts van Bueren JJ, van Ojik HH, Gerritsen AF (2004). Dual mode of action of a human antiepidermal growth factor receptor monoclonal antibody for cancer therapy. J. Immunol..

[57] Peipp M, Schneider-Merck T, Dechant M, Beyer T (2008). Tumor cell killing mechanisms of epidermal growth factor receptor (EGFR) antibodies are not affected by lung cancerassociated EGFR kinase mutations. J. Immunol..

[58] Lammerts van Bueren JJ, Bleeker WK, Brannstrom A, von Euler A (2008). The antibody zalutumumab inhibits epidermal growth factor receptor signaling by limiting intraand intermolecular flexibility. Proc. Natl. Acad. Sci. USA.

[59] Schlessinger J (2000). Cell signaling by receptor tyrosine kinases. Cell.

[60] Ferguson KM, Berger MB, Mendrola JM, Cho HS (2003). EGF activates its receptor by removing interactions that autoinhibit ectodomain dimerization. Mol. Cell.

[61] Fan Z, Lu Y, Wu X, Mendelsohn J (1994). Antibody-induced epidermal growth factor receptor dimerization mediates inhibition of autocrine proliferation of A431 squamous carcinoma cells. J. Biol. Chem..

[62] Fan Z, Masui H, Altas I, Mendelsohn J (1993). Blockade of epidermal growth factor receptor function by bivalent and monovalent fragments of 225 anti-epidermal growth factor receptor monoclonal antibodies. Cancer Res..

[63] Lammerts van Bueren JJ, Bleeker WK, Bogh HO, Houtkamp M (2006). Effect of target dynamics on pharmacokinetics of a novel therapeutic antibody against the epidermal growth factor receptor: Implications for the mechanisms of action. Cancer Res..

[64] Bastholt L, Specht L, Jensen K, Brun E (2007). Phase I/II clinical and pharmacokinetic study evaluating a fully human monoclonal antibody against EGFr (HuMax-EGFr) in patients with advanced squamous cell carcinoma of the head and neck. Radiother. Oncol..

[65] Spiridon CI, Ghetie MA, Uhr J, Marches R (2002). Targeting multiple Her-2 epitopes with monoclonal antibodies results in improved antigrowth activity of a human breast cancer cell line *in vitro* and *in vivo*. Clin. Cancer Res..

[66] Faderl S, Thomas DA, O'Brien S, Garcia-Manero G (2003). Experience with alemtuzumab plus rituximab in patients with relapsed and refractory lymphoid malignancies. Blood.

[67] Dechant M, Weisner W, Berger S, Peipp M (2008). Complement-dependent tumor cell lysis triggered by combinations of EGF-R antibodies. Cancer Res..

[68] Presta LG (2008). Molecular engineering and design of therapeutic antibodies. Curr. Opin. Immunol..

[69] Satoh M, Iida S, Shitara K (2006). Non-fucosylated therapeutic antibodies as next-generation therapeutic antibodies. Expert Opin. Biol. Ther..

[70] D'Amore F, Radford J, Jerkeman M, Relander T (2007). Zanolimumab (HuMax-CD4), a fully human monoclonal antibody: Efficacy and safety in patients with relapsed or treatment refractory non-cutaneous CD4^+^ T-cell lymphoma. Blood.

[71] Hagenbeek A, Gadeberg O, Johnson P (2008). First clinical use of ofatumumab, a fully human anti-CD20 monoclonal antibody in relapsed or refractory follicular lymphoma: results of a phase I/II trial. Blood.

